# 12-Month prevalence of known diabetes mellitus in Germany

**DOI:** 10.17886/RKI-GBE-2017-017

**Published:** 2017-03-15

**Authors:** Christin Heidemann, Ronny Kuhnert, Sabine Born, Scheidt-Nave Christa

**Affiliations:** Robert Koch Institute, Department for Epidemiology and Health Monitoring, Berlin, Germany

**Keywords:** DIABETES MELLITUS, PREVALENCE, HEALTH SURVEY, GERMANY, ADULTS

## Abstract

Diabetes mellitus is a metabolic disease involving chronic dysfunction of blood sugar regulation; if left untreated, it can result in serious secondary illnesses. In 2014 and 2015, a total of 7.0% of women and 8.6% of men in Germany with an age of 18 and over reported having diabetes mellitus in the past 12 months (these figures do not include gestational diabetes). There are significant differences in the 12-month prevalence among adults: the prevalence of known diabetes increases significantly with age, and is particularly high among people with a low educational status and those living in Saxony-Anhalt or Brandenburg. The Robert Koch Institute is currently developing a diabetes surveillance system in order to establish a data-based fundament for guiding health policy decisions in Germany.

## Introduction

Diabetes mellitus is a metabolic disorder that involves a dysfunction of blood glucose regulation [1]. It can cause chronically elevated blood sugar concentrations, which, if left untreated, can damage blood vessels and nerves. In turn, these disorders increase the risk of concomitant diseases and secondary illnesses such as heart attacks, strokes, renal dysfunction, retinal damage and diabetic foot syndrome [2]. Diabetes also reduces a person’s quality of life and life expectancy [3, 4]. Further, it results in high direct and indirect costs to the health system due to the expenses incurred through diabetes care and treatment as well as through the loss of resources associated with incapacities to work or forced early retirement [5].

The most important forms of diabetes are type 1, type 2 and gestational diabetes [6]. Type 1 diabetes is an autoimmune disease and mainly develops during childhood or adolescence. Type 2 diabetes is the most common form and usually affects adults over the age of 40. Alongside a genetic predisposition, the main risk factors linked to type 2 diabetes are an unfavourable diet, a lack of exercise, and the resulting overweight. Gestational diabetes normally only exists during pregnancy; however, it is linked to an increased risk of type 2 diabetes in older age [7].

## Indicator

The 12-month prevalence of diabetes mellitus for GEDA 2014/2015–EHIS was assessed using a self-administered paper-based or online questionnaire. The study posed the question ‘During the past 12 months, have you had any of the following diseases or conditions?’ This question was followed by a list of diseases that also included ‘diabetes (not including gestational diabetes)’. Thus, the study excludes gestational diabetes. However, it is not possible to differentiate between type 1 and type 2 diabetes in the results.

**GEDA 2014/2015-EHIS**

**Table:** 

**Data holder:**	Robert Koch Institute
**Aims:**	to provide reliable information about the population’s health status, health-related behaviour and health care in Germany, with the possibility of a European comparison
**Method:**	questionnaires completed on paper or online
**Population:**	people aged 18 years and above with permanent residency in Germany
**Sampling:**	registry office sample; randomly selected individuals from 301 communities in Germany were invited to participate
**Participants:**	24,016 people (10,872 men; 13,144 women)
**Response rate:**	26.9%
**Study period:**	November 2014 – July 2015
**Data protection:**	all participants were informed about the study’s aims and content and about data protection, and provided their informed consent

More information is available at

www.geda-studie.de

The present analysis is based on data provided by 23,345 participants aged 18 and above from the GEDA 2014/2015-EHIS study who also provided information about their 12-month prevalence of diabetes (671 participants were excluded from the analysis as this data was lacking). The analysis was conducted using a weighting factor that corrects for deviations within the sample from the population structure for gender and age within Germany’s federal states (as of 31 December 2014) as well as for municipality type and level of education. The article entitled German Health Update: New data for Germany and Europe [8], which is also published in this issue, sets out the details of the methodology employed for GEDA 2014/2015-EHIS. Educational status was defined in accordance with the International Standard Classification of Education (ISCED) [9]. P-values of <0.05 were considered statistically significant.

## Results and discussion

In GEDA 2014/2015-EHIS, 7.7% of participants aged 18 or above reported the presence of a diabetes mellitus (not including gestational diabetes) during the last 12 months (see Table 1). The prevalence was lower among women with 7.0% than among men with 8.6%.

The last telephone survey conducted by the Robert Koch Institute of adults aged 18 or over was undertaken in 2012 (GEDA 2012). This study found a comparable 12-month prevalence (7.7% overall) to that of GEDA 2014/2015-EHIS [10]. However, a comparison of gender-specific prevalence demonstrates small deviations in opposite directions. The 12-month prevalence derived from the GEDA 2012 data is slightly higher among women (7.5%) and slightly lower among men (7.9%) than the prevalence estimated by the current GEDA 2014/2015-EHIS data [10]. These variations are probably mostly due to the different questions posed by the studies. In GEDA 2012 respondents were asked whether they ever had a physician-diagnosed diabetes, which sustained during the preceding 12 months; this could explain the lower prevalence. However, the question did not exclude gestational diabetes; this could explain the higher prevalence of diabetes only among women. Data from the German Health Interview and Examination Study for Adults (DEGS1, 2008-2011) provides an indication of the degree of gestational diabetes occurring among women: the study found a prevalence of physician-diagnosed diabetes which had only occurred during pregnancy in women between 18 and 79 years of age of 1.2%. This corresponds to 16.3% of the lifetime prevalence of diabetes in women [11].

With respect to changes over time, a significant increase in lifetime prevalence of physician-diagnosed diabetes in adults of 18 and above is evident from the telephone surveys conducted in 2003 (GSTel03) compared with 2009 (GEDA 2009). During this time, the prevalence increased from 6.8% to 9.3% in women and from 5.4% to 8.2% in men [10, 12]. Similarly, a comparison of the examination surveys conducted between 1997 and 1999 (GNHIES98) and between 2008 and 2011 (DEGS1) demonstrates a significant increase in the lifetime prevalence of physician-diagnosed diabetes among 18- to 79-year-olds [11]. In addition, data from statutory health insurers also indicate an increased prevalence of diagnosed diabetes between 2000 and 2010 [13-15]. In contrast, no other relevant changes in prevalence were observed based on the telephone surveys conducted between 2009 and 2012 (GEDA 2009, GEDA 2010 and GEDA 2012). A slight trend towards an increased lifetime and 12-month prevalence was observed among men between 2009 and 2012; however, this trend in men was not statistically significant, nor was it observed in women (lifetime prevalence: 8.2%, 8.5%, 8.7% for men, 9.3%, 8.8%, 9.0% for women [10]; 12-month prevalence: 7.2%, 7.6%, 7.9% for men, 7.5%, 7.1%, 7.5% for women [10, 16, 17]).

Several factors need to be taken into account when interpreting these temporal developments in the prevalence of known diabetes. First, up to about one third of the increase in prevalence that occurred within the time period between 2000 and 2010 can be explained by population aging [11-13]. However, even after aging has been accounted for, the increase in known diabetes remains statistically significant [11, 12]. Second, the increase could also partially be explained by earlier diabetes diagnosis resulting from a stronger focus on diabetes among medical staff (such as since the introduction of the Disease Management Program for type 2 diabetes) [18]) or changes to diagnostic criteria [19, 20]. This would cause the proportion of diagnosed cases to rise, and the share of undetected cases to fall. The recently observed decrease in the prevalence of undiagnosed diabetes suggests that improvements may have resulted in an earlier detection of diabetes [21]. Third, improved care for people with diabetes (such as since the introduction of the Disease Management Program [18] and the module for the German National Disease Management Guideline (NDMG) on type 2 diabetes [22]) and the associated longer survival could also contribute to the increased prevalence of diagnosed diabetes. The increased proportions of 45- to 79-year-olds who are affected by diabetes but who have achieved their therapy goal with regard to the laboratory parameter HbA1c, who self-monitor their blood-glucose level or who have had annual eye and foot examination suggest that diabetes care has at least partly improved [23]. Finally, the temporal developments in behavioural risk factors continue to play a role in the dynamics of diabetes prevalence. However, diverging temporal trends have been observed for single risk factors what complicates the evaluation of changes in the overall diabetes risk. For example, the prevalence of physical inactivity decreased, whereas the prevalence of obesity increased [24, 25].

Analyses of the GEDA 2014/2015-EHIS data stratified according to age and level of education reveal significant differences in the 12-month prevalence of known diabetes among the adult population in Germany. The 12-month prevalence for men and women under 45 is no more than 2.0%; however, the prevalence rises strongly with age to 5.2% among women and 9.3% among men of 45 to 64 years of age, and to 17.6% among women and 21.1% among men of 65 and above. Moreover, men and women with a lower level of education are more likely to have known diabetes than those with a higher level of education. Whereas this difference is pronounced among women of all ages, it does not occur among men until the 45-to-64 age group (see Table 1). Similarly, a significantly higher prevalence of undetected diabetes was also observed with increasing age and lower educational status [21, 26].

**Fig. 1:**
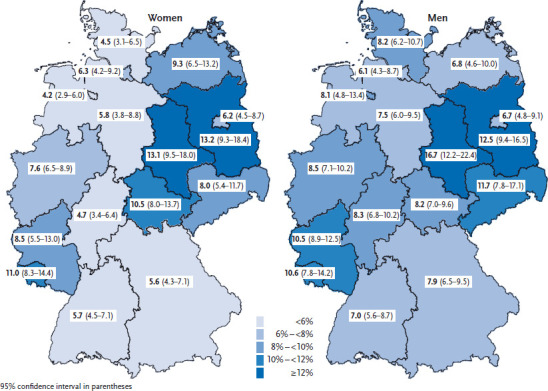
12-month prevalence of known diabetes (not including gestational diabetes) in women and men according to German federal state (N=23,345) Source: GEDA 2014/2015-EHIS

Compared to the average 12-month prevalence of known diabetes over all federal states, significantly higher prevalence estimates were observed among women in Saxony-Anhalt, Brandenburg, Thuringia and Saarland, and among men in Saxony-Anhalt, Brandenburg and Rhineland-Palatinate (see Figure 1). In contrast, women in Bremen, Schleswig-Holstein, Hessen, Baden-Württemberg and Bavaria, and men in Hamburg and Baden-Württemberg have significantly lower prevalence estimates compared to the national average. Even after the differences in age structure and educational status between the German federal states have been taken into account, most of the deviations from the national average remain (with the exception of men in Rhineland-Palatinate, Hamburg and Baden-Württemberg). Similar regional patterns were identified for the prevalence of physician-diagnosed diabetes by a pooled analysis of GEDA data from 2009, 2010 and 2012 and for the prevalence of diagnosed type 2 diabetes in an analysis of AOK health insurance data from 2010 [27, 28]. Nevertheless, it is important to remember that the prevalence of known diabetes may also differ considerably within a federal state [14, 29]. The comparison of EHIS data at the European level that was undertaken for the OECD/EU report ‘Health at a Glance: Europe 2016’ as well as for the article Health monitoring and health indicators in Europe that is also published in this issue shows that the 12-month prevalence of known diabetes in Germany is similar to the European average [30, 31]. When interpreting the different prevalence levels of known diabetes within Germany and Europe, alongside differences in age structure, differences in factors mentioned above such as the relation of diagnosed cases to undetected cases, the diabetes care situation, and risk factor load again need to be taken into account.

In summary, diabetes mellitus is a frequent disease among the adult German population with a particular high prevalence among women and men with an age of 45 or above, with a lower level of education, and from Saxony-Anhalt and Brandenburg. These population groups need to be more strongly targeted by measures aimed at prevention, early detection and care. The Robert Koch Institute is developing a diabetes surveillance system in order to establish a sustainable diabetes reporting and a data-driven basis for guiding health-policy decisions in Germany. This includes the definition of suitable indicators for tracking the risk factors and the burden associated with diabetes as well as the identification of existing barriers to data usage and of data gaps. Concept and current status of the diabetes surveillance system are described in detail in a Concepts & Methods article published in this issue [32].

## Key statements

- 7.7% of adults in Germany (7.0% of women and 8.6% of men) reported having diabetes mellitus (not including gestational diabetes) in the last 12 months.
- Men and women in Saxony-Anhalt and Brandenburg have a significantly higher 12-month prevalence of known diabetes compared to the average prevalence over all federal states in Germany.
- After the age of 45, the 12-month prevalence for known diabetes rises dramatically with increasing age among both men and women.
- Adults with a lower level of education more frequently reported having diabetes in the last 12 months than adults with a higher level of education.

## Figures and Tables

**Table 1:** 12-month prevalence (95% CI) of known diabetes (not including gestational diabetes) according to gender, age and educational status (N=23,345*) Source: GEDA 2014/2015-EHIS

**Women**	**%**	**(95%-CI)**	**Men**	**%**	**(95%-CI)**
**Women total**	**7.0**	**(6.4-7.6)**	**Men total**	**8.6**	**(7.9-9.2)**
**18–29 Years** Low education Medium education High education	1.1 2.7 0.6 0.5	(0.6-1.9) (1.1-6.5) (0.3-1.2) (0.1-1.6)	**18 – 29 Years** Low education Medium education High education	0.5 0.3 0.6 0.5	(0.2-1.0) (0.1-1.3) (0.2-1.4) (0.1-3.3)
**30 – 44 Years** Low education Medium education High education	1.4 3.7 1.3 0.4	(0.9-2.1) (1.7-8.1) (0.8-2.2) (0.2-1.0)	**30 – 44 Years** Low education Medium education High education	2.0 2.0 2.3 1.6	(1.4-2.9) (0.7-5.5) (1.4-3.7) (0.9-2.9)
**45 – 64 Years** Low education Medium education High education	5.2 8.5 5.0 3.1	(4.5-6.1) (6.3-11.3) (4.0-6.1) (2.2-4.1)	**45 – 64 Years** Low education Medium education High education	9.3 16.9 9.7 5.9	(8.2-10.6) (13.0-21.7) (8.1-11.7) (4.8-7.3)
**≥ 65 Years** Low education Medium education High education	17.6 20.5 15.6 15.9	(15.9-19.6) (17.5-23.8) (13.3-18.4) (11.9-20.8)	**≥ 65 Years** Low education Medium education High education	21.1 24.0 21.3 19.5	(19.1-23.2) (19.7-28.9) (18.4-24.5) (16.6-22.6)
**Total (women and men)**	**7.7**	**(7.3-8.2)**	**Total (women and men)**	**7.7**	**(7.3-8.2)**

*N = 52 missing values for educational status; CI = confidence interval
